# The role of FOXD2-AS1 in cancer: a comprehensive study based on data mining and published articles

**DOI:** 10.1042/BSR20190372

**Published:** 2020-11-13

**Authors:** Yongping Zhang, Chaojie Liang, Yu Zhang, Zhinmin Wang, Ruihuan Li, Zhigang Wei, Jiansheng Guo

**Affiliations:** Department of General Surgery, First Hospital/First Clinical College of Shanxi Medical University, Taiyuan 030001, China

**Keywords:** FOXD2-AS1, long non-coding RNA, meta-analysis, Neoplasm, prognosis

## Abstract

**Background and aims:** Long non-coding RNA (lncRNA) FOXD2 adjacent opposite strand RNA 1 (FOXD2-AS1) is aberrantly expressed in various cancers and associated with cancer progression. A comprehensive meta-analysis was performed based on published literature and data in the Gene Expression Omnibus database, and then the Cancer Genome Atlas (TCGA) dataset was used to assess the clinicopathological and prognostic value of FOXD2-AS1 in cancer patients.

**Methods:** Gene Expression Omnibus databases of microarray data and published articles were used for meta-analysis, and TCGA dataset was also explored using the GEPIA analysis program. Hazard ratios (HRs) and pooled odds ratios (ORs) with 95% confidence intervals (CIs) were used to assess the role of FOXD2-AS1 in cancers.

**Results:** This meta-analysis included 21 studies with 2391 patients and 25 GEO datasets with 3311 patients. The pooled HRs suggested that highly expressed FOXD2-AS1 expression was correlated with poor overall survival (OS) and disease-free survival (DFS). Similar results were obtained by analysis of TCGA data for 9502 patients. The pooled results also indicated that FOXD2-AS1 expression was associated with bigger tumor size and advanced TNM stage, but was not related to age, gender, differentiation and lymph node metastasis.

**Conclusion:** The present study demonstrated that FOXD2-AS1 is closely related to tumor size and TNM stage. Additionally, increased FOXD2-AS1 was a risk factor of OS and DFS in cancer patients, suggesting FOXD2-AS1 may be a potential biomarker in human cancers.

## Introduction

Malignant tumors pose a great threat to human health [1]. Each year, there are approximately 14 million new cases of malignant tumors worldwide and more than 8.2 million deaths [2,3]. The prognosis for cancers is still poor, the difficulties of early cancer diagnosis and the lack of tumor-specific targeted drugs is the main reason [4]. Therefore, there is an urgent need for the identification of tumor-specific diagnostic biomarkers.

Long non-coding RNA (lncRNA) was originally discovered during large-scale sequencing of mouse full-length complementary DNA (cDNA) libraries [5], are RNA molecules over 200 nt in length that cannot be translated into proteins [6]. LncRNA was initially considered as noise, but the development of high-throughput sequencing and gene chip technology has revealed that many lncRNAs are abnormally expressed in tumor tissues. These lncRNAs are closely related to tumor resistance, cancer development, invasion and metastasis, suggesting that lncRNAs may be a new class of predictors or therapeutic targets for cancers [7,8]. Some lncRNAs have been identified as prognostic biomarkers for cancer patients, including HOTAIR [9], CRNDE [10], ZEB1-AS1 [11], and PCAT-1 [12].

LncRNA FOXD2 adjacent opposite strand RNA 1 (FOXD2-AS1) is located at chromosome 1p33, and has been linked to deterioration and progression of cancers. FOXD2-AS1 is elevated in several cancers, such as nasopharyngeal carcinoma (NC) [13], hepatocellular carcinoma (HCC) [14–17], gastric cancer (GC) [18], colorectal cancer (CRC) [19,20], non-small cell lung cancer (NSCLC) [21,22] and esophageal squamous cell carcinoma (ESCC) [22–25], breast cancer [26], glioma [27–30] and so on. The overexpression of FOXD2-AS1 has also been associated with clinicopathological characteristics and prognosis of cancers. However, the association between FOXD2-AS1 expression and clinicopathological characteristics in cancers remains controversial, and most studies have been limited by small sample size. Su et al. [31] reported that high FOXD2-AS1 expression was associated with T stage and recurrence, but not with lymph node metastasis and differentiation, and overexpression of FOXD2-AS1 was related to poor overall survival (OS) and disease-free survival (DFS) in bladder cancer. Xu et al. [18] found that FOXD2-AS1 expression was related to tumor size, TNM stage, and lymphatic metastasis, but not to gender, age and differentiation, and overexpression of FOXD2-AS1 was correlated with a high risk of DFS in GC. Bao et al. [32] found no relationship between FOXD2-AS1 and clinicopathological characteristics, but observed that elevated FOXD2-AS1 expression was associated with a poor OS and DFS in ESCC. Interestingly, Ren et al. [33] reported that FOXD2-AS1 was related to Clark level and distant metastasis, however, FOXD2-AS1 was not related to OS or DFS. To date, there has been a meta-analysis about the FOXD2-AS1, however, the studies included were limited [34], so we performed a comprehensive meta-analysis based on GEO datasets and published articles, and assessed the the Cancer Genome Atlas (TCGA) dataset to analysis the clinicopathological and prognostic value of FOXD2-AS1 in patients in pan-cancers.

## Materials and methods

### Search strategy and study selection

PubMed, Web of Science, and EMBASE databases were searched for published articles, and FOXD2-AS1 microarray data were extracted from GEO profiles (http://www.ncbi.nlm.nih.gov/geoprofiles/) and GEO datasets (http://www.ncbi.nlm.nih.gov/gds/). Only GPL570 platform data were used (Affymetrix Human Genome U133 Plus 2.0 Array, HG-U133_Plus_2) to minimize impacts on heterogeneity in later analyses. The databases were searched up to 1 January 2019. The key search words were ‘FOXD2-AS1’ OR ‘Long noncoding RNA FOXD2-AS1’ OR ‘LncRNA FOXD2-AS1’ AND ‘cancers’ or ‘neoplasm’.

We set the inclusion criteria for articles in this meta-analysis as follows: (1) use of qRT-PCR or RNA-seq data to measure the expression of FOXD2-AS1 in tumor tissues; (2) reported association between FOXD2-AS1 expression and clinicopathological characteristics and prognosis; and (3) reported specific hazard ratios (HRs) with 95% confidence interval (CI) or inclusion of sufficient data so that these parameters can be calculated by survival curves.

The exclusion criteria were: (1) conference reports, case reports, reviews, letters, and editorials; (2) studies that only reported the molecular function of FOXD2-AS1; (3) non-human studies in articles; and (4) duplicate articles.

### Data extraction and quality assessment

Two investigators (Chaojie Liang and Yongping Zhang) performed the search independently and the identified articles were assessed based on the criteria. The extracted data included clinicopathologcial characteristics, OS, and DFS. Newcastle–Ottawa Scale (NOS) criteria [35] were used to assess the quality of studies. NOS score ≥ 6 was considered high-quality studies, otherwise, the studies were considered as low-quality.

### Public data and tools

A web program named GEPIA was used to analyze the relationship between FOXD2-AS1 and prognosis. In GEPIA, one-way ANOVA was used to analyze the expression of FOXD2-AS1, and the Kaplan–Meier method and the log-rank test were used to calculate survival analysis, and the cut-off values were analyzed by GEPIA.

### Statistical analyses

Statistical data were analyzed by STATA14.2 software. We extracted the HR value with 95% CI from survival curve data by Engauge Digitizer 10.0. Pooled ORs with 95% CIs were calculated for the association of FOXD2-AS1 expression and clinicopathological features. HRs with 95% CIs were calculated to assess the correlation between FOXD2-AS1 expression and prognosis. Heterogeneity was assessed by *I^2^* test and Q test, and the random effect was performed if the *I^2^* > 50%, and when the *I^2^* < 50%, fixed effect was used. We considered the results significant when the pooled OR or HR values with 95% CI did not overlap 1. Sensitivity analysis or subgroup analysis was performed to analyze the presence of heterogeneity and stability of results, and publication bias was assessed by Begg’s funnel.

## Results

### Study identification and characteristics

**Figure 1:**
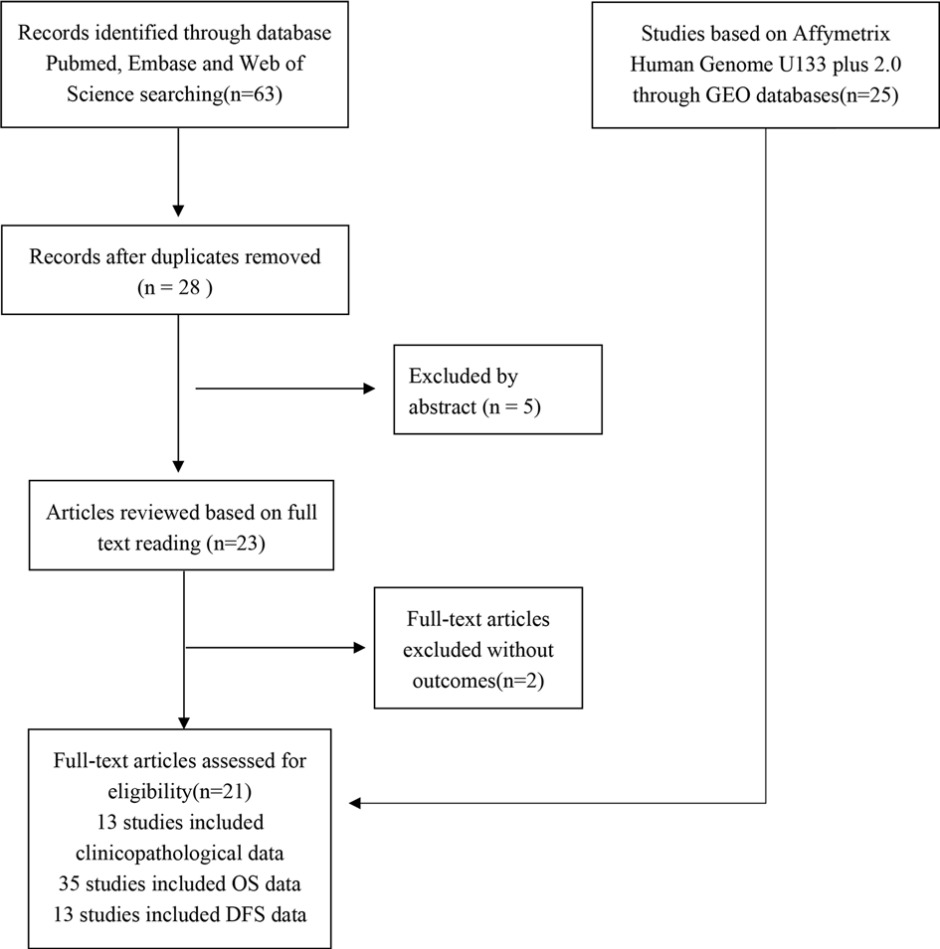
Flow diagram of the meta-analysis

The screening process employed is shown in Figure 1. Twenty-one studies [13,14,16–18,20,21,23,29,31,33,36–45] with a total of 2391 patients were selected. The selected studies included four HCC study, two colorectal cancer (CRC) study, one ESCC study, one tongue squamous cell carcinoma study, one GC study, one NC study, one bladder cancer (BC) study, two glioma studies, one NSCLC study, two cutaneous melanoma (CM) study, and two papillary thyroid carcinoma (PTC) study, one cervical carcinoma (CC) study, one head and neck carcinoma study (HNSC) and one osteosarcoma study (OSC). The studies were selected for inclusion in this meta-analysis based on the inclusion and exclusion criteria. These articles were published from 2017 to 2020, the sample size ranged from 50 to 481 patients, and all studies were from China and published in English or Chinese. All studies scored >6 on the NOS, which indicated that all the studies were of high quality. The details of articles are summarized in Table 1.

**Table 1:** Characteristics of studies included in the meta-analysis

Study	Year	Country	Sample size	Tumor type	Cut-off value	Laboratory method	Gender male (Y/N) female (Y/N)	Age old (Y/N) young (Y/N)	Tumor size: big (Y/N) small (Y/N)	Differentiation low (Y/N) high and moderate (Y/N)	Lymph node metastasis yes (Y/N) no (Y/N)	UICC stage I, II (Y/N) III, IV (Y/N)	Survival information	HR	NOS score
Chen [13]	2017	China	50	NC	Median	qRT-PCR	NA	NA	NA	NA	NA	NA	OS	2.69 (1.17–5.31) (C)	6
															
Bao [23]	2017	China	147	ESCC	Median	qRT-PCR	56/60	31/35	15//10	19/13	31/35	31/35	OS	1.94 (1.17–3.06) (R)	8
							17/14	42/39	58/64	54/61	42/29	42/39	DFS	2.71 (1.53–4.80) (R)	
Rong [21]	2017	China	35	NSCLC	NA	qRT-PCR	16/8	11/4	12/5	8/5	21/7	21/7	OS	3.12 (2.29–5.56) (R)	8
							8/3	13/7	12/6	3/19	2/4	2/4			
Dong [36]	2018	China	124	Glioma	NA	qRT-PCR	36/40	32/30	29/22	34/52	NA	NA	OS	3.56 (1.48–5.72) (R)	8
							24/24	28/34	31/42	26/12					
Shen [29]	2018	China	29	Glioma	Median	qRT-PCR	NA	NA	NA	NA	NA	NA	OS	1.53 (1.02–3.96) (C)	6
															
Su [31]	2018	China	84	BC	Median	qRT-PCR	34/34	20/16	NA	6/9	14/10	14/10	OS	2.32 (1.07-5.31) (C)	7
							8/8	22/26		36/33	28/32	28/32	DFS	2.12 (1.07–5.31) (C)	
Ren [33]	2018	China	124	CM	NA	qRT-PCR	32/34	35/32	NA	NA	NA	NA	NA	NA	7
							30/28	27/30							
Xu [18]	2018	China	106	GC	Median	qRT-PCR	31/35	27/30	35/20	37/38	37/36	37/36	DFS	2.28 (1.30–5.78) (R)	8
							22/18	26/23	18/33	16/15	16/27	16/27			
Zhang [37]	2018	China	84	PTC	NA	qRT-PCR	NA	NA	NA	NA	NA	NA	OS	1.65 (1.07–3.96) (C)	6
															
Chang [14]	2018	China	360	HCC	NA	qRT-PCR	NA	NA	NA	NA	NA	NA	OS	1.63 (1.16–2.52) (R)	8
															
Zhu [32]	2018	China	481	CRC	Median	qRT-PCR	NA	NA	NA	NA	NA	NA	OS	1.69 (1.17–2.45) (C)	6
															
Chen [39]	2019	China	70	CM	Median	qRT-PCR	NA	NA	NA	NA	NA	NA	OS	3.332 (1.03–6.09) (R)	6
															
Lei [16]	2019	China	88	HCC	NA	qRT-PCR	NA	NA	NA	NA	NA	NA	OS	1.96 (1.02–3.96) (C)	6
															
Li [40]	2019	China	160	PTC	Median	qRT-PCR	50/37	28/28	25/27	NA	50/23	35/39	OS	2.043 (1.579–3.01) (C)	8
							36/31	64/40	67/41		42/45	57/29			
Ren [41]	2019	China	35	OSC	Median	qRT-PCR	7/8	NA	15/7	NA	NA	5/9	OS	3.06 (1.03–6.98) (C)	6
							11/9		3/10			13/8			
Zhang [20]	2019	China	60	CRC	Median	qRT-PCR	NA	NA	NA	NA	NA	NA	OS	2.245 (1.01–4.32) (C)	6
															
Xu [42]	2019	China	105	HCC	Median	qRT-PCR	14/15	23/21	26/14	17/19	NA	NA	NA	NA	6
							22/21	13/15	10/22	19/17					
Chen [43]	2019	China	85	HNSC	NA	qRT-PCR	28/22	21/19	NA	NA	NA	18/28	NA	NA	6
							16/19	23/22				25/13			
Dou [44]	2020	China	63	CC	Median	qRT-PCR	NA	17/19	11/13	22/17	19/9	NA	OS	1.73 (1.07–4.52) (C)	7
								15/12	21/18	10/14	13/22				
Hu [17]	2020	China	60	HCC	Median	qRT-PCR	9/8	11/9	19/9	16/5	NA	12/26	NA	NA	6
							20/23	22/18	12/20	15/24		12/10			
Zhou	2020	China	41	TSCC	Median	qRT-PCR	16/15	11/13	NA	6/1	18/5	1/5	NA	NA	8
							5/5	10/7		15/19	3/15	20/5			

Abbreviations: C, HR was estimated by curve; N, no; R, HR was reported; Y, yes.

As shown in Tables 2 and 3, 19 GEO databases with 2265 patients were included in this meta-analysis for OS. There were 11 studies from the United States, 14 studies from Western countries, and 6 studies from Asia. Studies of nine different types of tumors were included in the meta-analysis including lung cancer (*n*=6), colon cancer (*n*=3), breast cancer (*n*=3), ovarian cancer (*n*=2), diffuse large B-cell lymphoma (DLBCL, *n*=1), chronic lymphocytic leukemia (CLL, *n*=1), glioblastoma (GBM, *n*=1), meningioma (*n*=1), and melanoma (*n*=1). We also analyzed ten GEO datasets containing records for 1568 patients to calculate DFS. This analysis included three kind of cancers: colon cancer (*n*=5), breast cancer (*n*=3), and lung cancer (*n*=2).

**Table 2:** OS characteristics of studies based on Affymetrix Human Genome U133 Plus 2.0

Type of cancer	GEO number	Year	Country	Number of patients	Outcome measure	Follow-up (month)	Cut-off value	HR
Lung cancer	GSE3141	2005	U.S.A.	111	OS	87	Median	1.426 (0.847–2.402)
Colon cancer	GSE17536	2009	U.S.A.	177	OS	142	Median	1.043 (0.657–1.647)
Colon cancer	GSE17538	2009	U.S.A.	232	OS	142	Median	1.326 (0.982–1.992)
CLL	GSE22762	2011	Germany	107	OS	72	Median	2.121 (0.935–4.816)
Lung cancer	GSE30129	2011	France	293	OS	256	Median	1.163 (0.876–1.543)
Lung cancer	GSE31210	2011	Japan	226	OS	128	Median	1.239 (0.738–2.404)
Lung cancer	GSE37745	2012	Sweden	196	OS	187	Median	0.978 (0.704–1.358)
Lung cancer	GSE50081	2013	Canada	181	OS	144	Median	1.458 (0.926–2.298)
Breast cancer	GSE58812	2015	France	107	OS	169	Median	1.491 (0.719–3.09)
GBM	GSE7696	2008	Switzerland	80	OS	72	Median	1.238 (0.754–2.032)
Meningioma	GSE16581	2010	U.S.A.	67	OS	11	Median	2.073 (0.659–7.683)
Melanoma	GSE19234	2009	U.S.A.	44	OS	186	Median	1.574 (0.741–3.865)
Ovarian cancer	GSE19829	2010	U.S.A.	28	OS	115	Median	1.473 (0.829–4.105)
Breast cancer	GSE20711	2011	Canada	88	OS	14	Median	1.108 (0.602–2.441)
DLBCL	GSE23501	2010	U.S.A.	69	OS	72	Median	1.468 (0.792–4.383)
Lung cancer	GSE29013	2011	U.S.A.	55	OS	82	Median	1.282 (0.8031–3.264)
Colon cancer	GSE29623	2014	U.S.A.	65	OS	120	Median	1.151 (0.725–2.528)
Ovarian cancer	GSE30161	2012	U.S.A.	58	OS	127	Median	1.051 (0.643–2.036)
Breast cancer	GSE48390	2014	Taiwan	81	OS	69	Median	1.234 (0.877–4.035)

**Table 3:** DFS characteristics of studies based on Affymetrix Human Genome U133 Plus 2.0

Type of cancer	GEO number	Year	Country	Number of patients	Outcome measure	Follow-up (month)	Cut-off value	HR
Colon cancer	GSE14333	2010	Australia	226	DFS	142	Median	1.432 (0.862–2.998)
Colon cancer	GSE17536	2009	U.S.A.	145	DFS	142	Median	1.409 (0.791–2.713)
Colon cancer	GSE17538	2009	U.S.A.	200	DFS	142	Median	1.28 (0.897–2.349)
Breast cancer	GSE21653	2010	France	252	DFS	189	Median	1.38 (0.884–2.154)
Lung cancer	GSE30219	2013	France	278	DFS	256	Median	1.528 (1.053–2.215)
Colon cancer	GSE38832	2014	U.S.A.	92	DFS	111	Median	1.02 (0.273–3.811)
Lung cancer	GSE50081	2013	Canada	177	DFS	144	Median	1.271 (0.733–2.205)
Breast cancer	GSE6532	2007	Canada	87	DFS	202	Median	1.948 (0.923–4.111)
Colon cancer	GSE29623	2014	U.S.A.	53	DFS	120	Median	1.894 (0.513–6.998)
Breast cancer	GSE61304	2005	Singapore	58	DFS	85	Median	1.335 (0.530–3.364)

### Prognostic value of FOXD2-AS1 for OS

**Figure 2:**
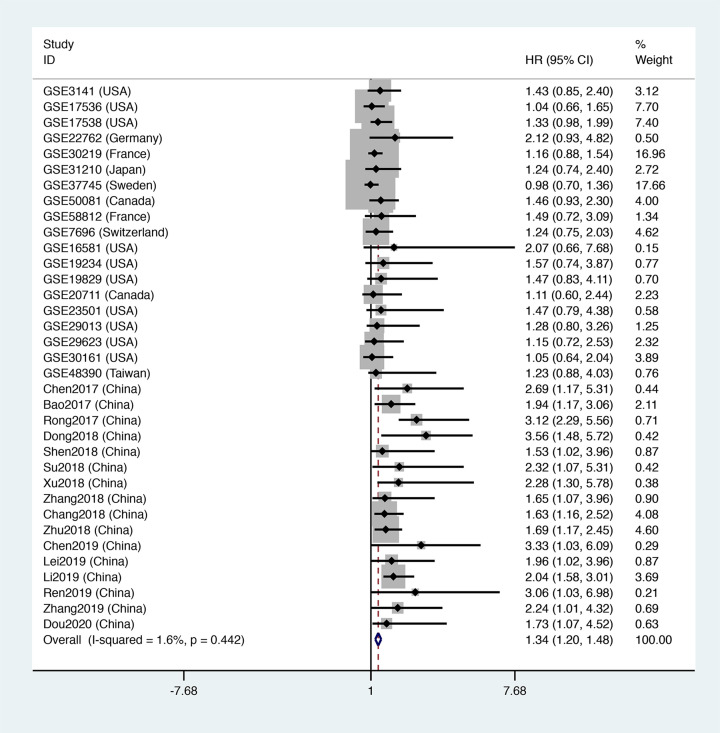
The relationship between FOXD2-AS1 expression and OS rate

**Figure 3:**
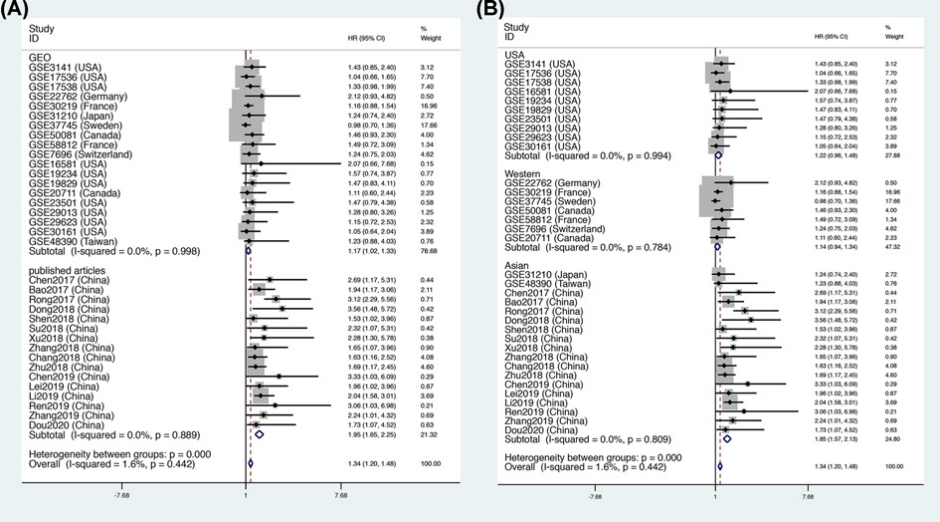
Subgroup analysis of OS Subgroup analysis by (**A**) source and (**B**) region.

**Figure 4:**
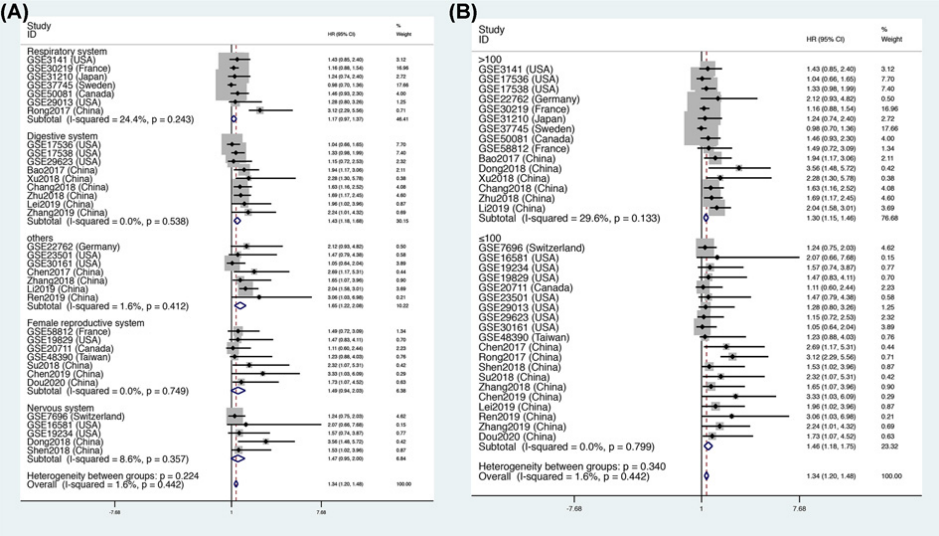
Subgroup analysis of OS Subgroup analysis by (**A**) tumor type and (**B**) sample size.

This meta-analysis included data for a total of 4241 patients. The pooled HR indicated that FOXD2-AS1 expression was closely related to a poor OS (HR = 1.34, 95% CI = [1.20, 1.48], *P*<0.001, Figure 2), and there was no significant heterogeneity (*I^2^* = 0). In addition, we performed subgroup analysis according to source, region, tumor type and tumor size, as shown in Table 4. The subgroup analysis for source demonstrated that FOXD2-AS1 expression was correlated with a high risk of OS in the GEO data (OS: HR = 1.17, 95% CI = [1.02, 1.33], *P*<0.05, Figure 3A) and published articles (OS: HR = 1.95, 95% CI = [1.65, 2.25], *P*<0.05, Figure 3A). Interestingly, the subgroup analysis for region revealed that the expression of FOXD2-AS1 was associated with poor OS only in Asian subjects (OS: HR = 1.85, 95% CI = [1.58, 2.13], *P*<0.05, Figure 3B), but not in U.S.A. subjects (OS: HR = 1.22, 95% CI = [0.96, 1.48], *P*>0.05, Figure 3B) or Western subjects (OS: HR = 1.14, 95% CI = [0.94, 1.34], *P*>0.05, Figure 3B). The subgroup analysis for tumor type demonstrated that elevated FOXD2-AS1 expression was associated with a poor OS in patients with digestive tumors (OS: HR = 1.43, 95% CI = [1.18, 1.68], *P*<0.05, Figure 4A) and other tumors (OS: HR = 1.65, 95% CI = [1.22, 2.08], *P*<0.05, Figure 4A), but not in the respiratory system (OS: HR = 1.17, 95% CI = [0.97, 1.37], *P*>0.05, Figure 4A), the female reproductive system (OS: HR = 1.47, 95% CI = [0.95, 3.08], *P*>0.05, Figure 4A), or the nervous system (OS: HR = 1.48, 95% CI = [0.95, 2.00], *P*>0.05, Figure 4A). When subgroup analysis was conducted according to sample size, the pooled HRs indicated that increased FOXD2-AS1 expression was associated with poor OS in both subgroups (OS: HR = 1.30, 95% CI = [1.14, 1.46], *P*<0.05, *n*>100, Figure 4B) (OS: HR = 1.34, 95% CI = [1.20, 1.49], *P*<0.05, *n*≤100, Figure 4B).

**Table 4:** Subgroup analysis of OS by data source, region, tumor type, sample size

Subgroups	Number of studies	Number of patients	Pooled HR (95% CI)	*P*_Het_	*I^2^*(%)	*P*-value
*Data source*						
Published articles	16	1976	1.95 (1.65, 2.25)	0.889	0.0	<0.05
GEO	19	2265	1.17 (1.02, 1.33)	0.998	0.0	<0.05
*Region*						
U.S.A.	10	906	1.22 (0.96–1.48)	0.994	0.0	>0.05
Western	7	498	1.14 (0.94–1.34)	0.784	0.0	>0.05
Asian	18	2837	1.85 (1.58–2.13)	0.442	0.0	<0.05
*Tumor type*						
Respiratory system	7	1097	1.17 (0.97–1.37)	0.243	24.4	>0.05
Digestive system	9	1716	1.43 (1.18–1.68)	0.538	0.0	<0.05
Others	7	563	1.65 (1.22–2.08)	0.412	1.6	<0.05
Female reproductive system	7	521	1.47 (0.95–3.08)	0.749	0.0	>0.05
Nervous system	5	344	1.48 (0.95–2.00)	0.357	8.6	>0.05
*Sample size*						
>100	15	3008	1.30 (1.14, 1.46)	0.133	29.6	<0.05
≤100	20	1233	1.34 (1.20, 1.49)	0.799	0.0	<0.05

Abbreviation: *n*, number of sample size.

### Prognostic value of FOXD2-AS1 for DFS

**Figure 5:**
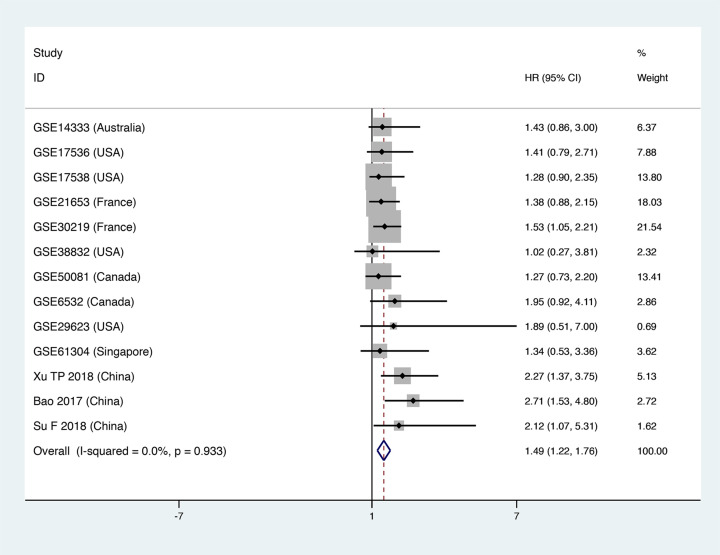
Forest plot of DFS

The prognostic value of FOXD2-AS1 for DFS of cancer patients was assayed using data that included 13 studies and 2007 patients; and we found a significant relationship between FOXD2-AS1 and DFS (HR = 1.49, 95% CI = [1.22, 1.76], *P*<0.05, Figure 5).

**Figure 6:**
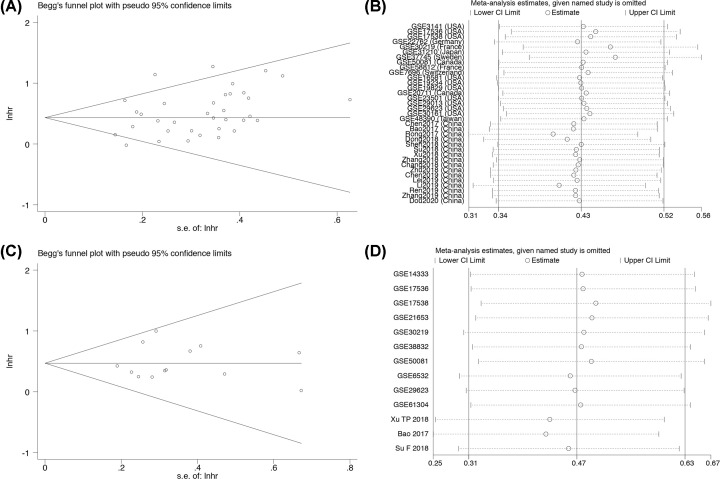
Begg’s publication bias plots and sensitivity analysis of studies evaluating the relationship between FOXD2-AS1 expression and survival rate (**A**) Begg’s publication bias of OS. (**B**) Sensitivity analysis of OS. (**C**) Begg’s publication bias of DFS. (D) Sensitivity analysis of DFS.

We performed Begg’s funnel plot analysis to assess potential publication bias. As shown in Figure 6, no significant publication bias was identified for OS (*P*=0.159, Figure 6A) or DFS (*P*=0.669, Figure 6C). Sensitivity analysis can assess the stability and reliability of meta-analysis results, and can also assess whether the combined results are affected by a single study by calculating the results when individual studies are omitted and determining if the result is within the CI. Sensitivity analysis was performed and the results are shown in Figure 6B,D, indicating the results were robust and reliable.

### Validation of TCGA dataset results

**Figure 7:**
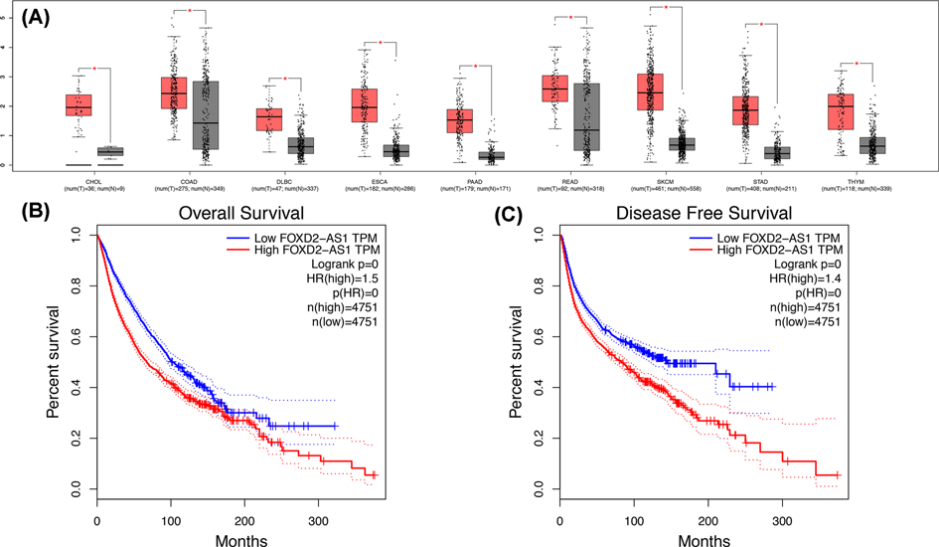
The expression of FOXD2-AS1 in TCGA dataset (**A**) FOXD2-AS1 expression in CHOL, COAD, DLBC, ESCA, PAAD, READ, SKCM, STAD, and THYM, which was analyzed by one-way ANOVA. ‘*’ means log2FC value > 1 and *P*-value <0.01. (**B**) OS rate of FOXD2-AS1 expression in TCGA (*n*=9502, Log-rank *P*<0.01). (**C**) DFS rate of FOXD2-AS1 in TCGA cohort (*n*=9502, Log-rank *P*<0.01). Red boxes indicate cancer, and gray boxes indicate normal.

**Figure 8:**
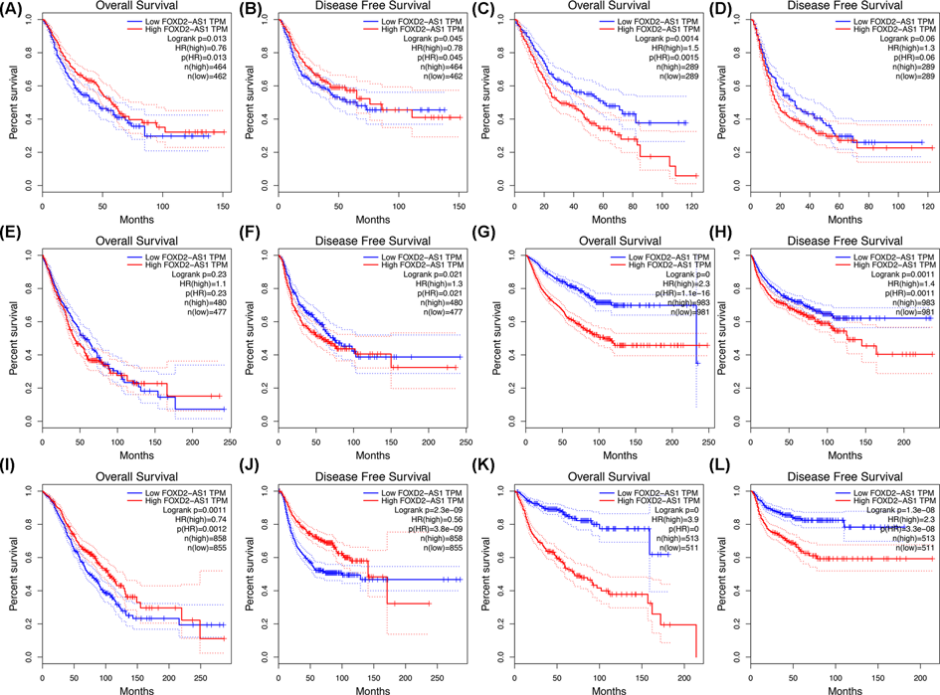
Validation of FOXD2-AS1 expression in TCGA cohort (**A**) OS in GI cancer patients (*n*=926, Log-rank *P*=0.013). (**B**) DFS in GI tumors (*n*=926, Log-rank *P*=0.045). (**C**) OS in hepatobiliary and pancreatic cancer patients (*n*=578, Log-rank *P*=0.0014). (**D**) DFS in hepatobiliary and pancreatic cancer patients (*n*=578, Log-rank *P*=0.06). (**E**) OS in respiratory cancer patients (*n*=957, Log-rank *P*=0.23). (**F**) DFS in respiratory cancer patients (*n*=957, Log-rank *P*=0.021). (**G**) OS in urinary cancer patients (*n*=1964, Log-rank <0.001). (**H**) DFS in urinary cancer patients (*n*=1964, Log-rank =0.001). (**I**) OS in female reproductive cancer patients (*n*=1703, Log-rank *P*=0.0011). (**J**) DFS in female reproductive cancer patients (*n*=1703, Log-rank *P*<0.001). (**K**) OS in head and neck cancer patients (*n*=1024, Log-rank *P*<0.001). (**L**) DFS in head and neck cancer patients (*n*=1024, Log-rank *P*<0.001).

Next, we explored FOXD2-AS1 expression in all cancer types using data from the TCGA dataset. As shown in Figure 7A, FOXD2-AS1 was overexpressed in cholangiocarcinoma (CHOL), colon adenocarcinoma (COAD), lymphoid neoplasm diffuse large B-cell lymphoma (DLBC), esophageal carcinoma (ESCA), pancreatic adenocarcinoma (PAAD), rectum adenocarcinoma (READ), skin cutaneous melanoma (SKCM), stomach adenocarcinoma (STAD), and thymoma (THYM), determining using a |log2FC| cutoff > 1 and a q-value < 0.01. A total of 9502 patients with digestive, respiratory, urinary, female reproductive, blood, and urinary systems cancers were included in the analysis. According to FOXD2-AS1 expression, the patients were divided into two groups according to mean expression by GEPIA. The results indicated that FOXD2-AS1 expression was correlated with a high risk of poor OS (Figure 7B) and DFS (Figure 7C). We also explored the prognostic role of FOXD2-AS1 in different tumor types, such as gastrointestinal (GI; Figure 8A,B), hepatobiliary, and pancreatic cancers (Figure 8C,D). As shown in Figure 8, FOXD2-AS1 expression was related to poor OS in hepatobiliary and pancreatic cancer (Figure 8C), urinary cancer (Figure 8G), and head and neck cancers (Figure 8K). However, no significant association was found between FOXD2-AS1 expression and OS in cancers of the respiratory system (Figure 8E). FOXD2-AS1 expression indicated poor DFS in urinary (Figure 8H), respiratory (Figure 8F), and head and neck tumors (Figure 8L), but FOXD2-AS1 expression was not related to DFS in hepatobiliary and pancreatic cancers (Figure 8D). Interestingly, the high expression of FOXD2-AS1 was related to favorable prognosis in GI (Figure 8A,B) and female reproductive cancers (Figure 8I,J).

### Association between FOXD2-AS1 and clinicopathological characteristics

**Figure 9:**
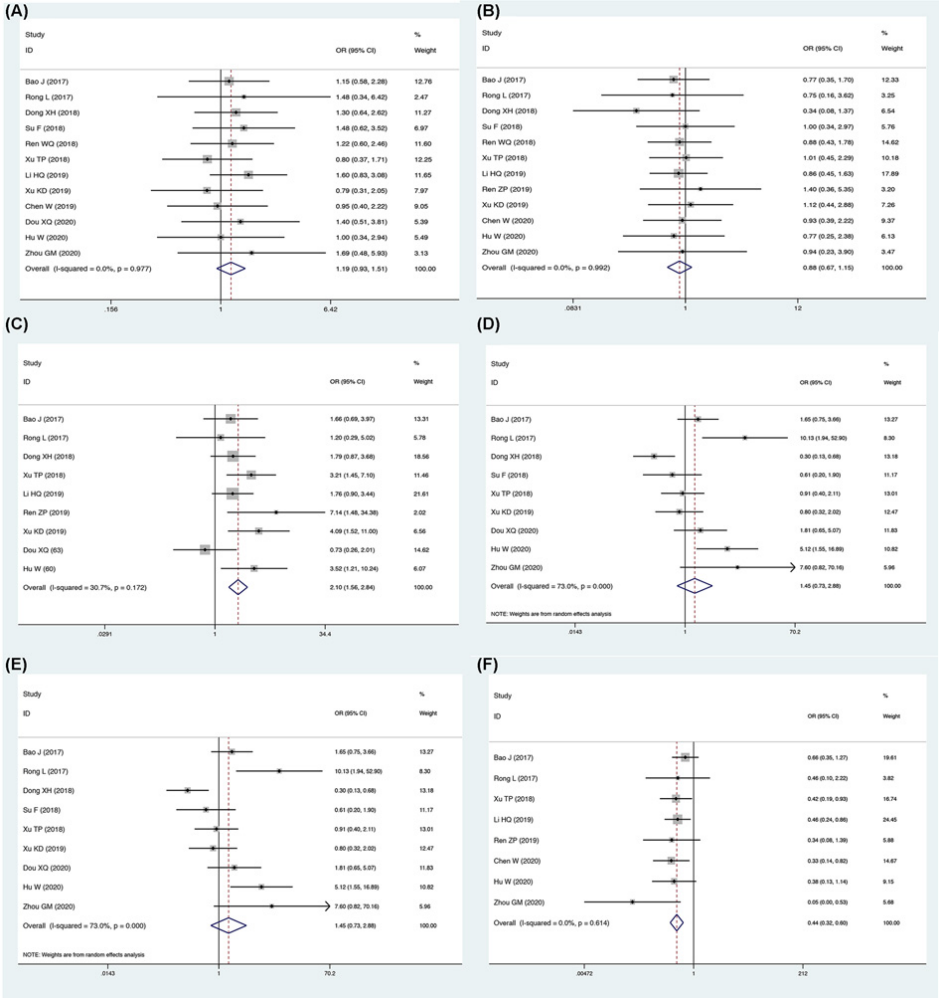
Meta-analysis evaluation of the association between FOXD2-AS1 expression and clinicopathological characteristics (**A**) Gender; (**B**) age; (**C**) tumor size; (**D**) differentiation; (**E**) lymph node metastasis; and (**F**) TNM stage.

The pooled ORs with 95% CI were calculated and are shown in Table 5. The pooled results indicated that high FOXD2-AS1 expression was significantly related to larger tumor size (bigger: small: OR = 2.01, 95% CI = [1.56, 2.84], *P*<0.001, Figure 9C), lymph node metastasis (yes: no, OR = 2.26, 95% CI = [1.22, 4.22], *P*<0.001, Figure 9E) advanced TNM stage (I+II: III+IV, OR = 0.44, 95% CI = [0.32, 0.60], *P*=0.012, Figure 9F). However, no significant relationship was identified between FOXD2-AS1 and gender (male: female, OR = 0.88, 95% CI = [0.67, 1.15], *P*=0992, Figure 9A), age (>60 vs ≤60, OR = 1.19, 95% CI = [0.93, 1.51], *P*=0.336, Figure 9B) and low differentiation (low: moderate+high, OR = 1.45, 95% CI = [0.73, 2.88], *P*=0283, Figure 9D). Begg’s funnel plot analysis showed that there was no publication bias for clinicopathological value (gender (*P*=0.707), age (*P*=0.452), tumor size (*P*=0.308), differentiation (*P*=0.806), or lymph node metastasis (*P*=0.308), or TNM stage (*P*=1)).

**Table 5:** LncRNA FOXD2-AS1 clinicopathological features for cancers

Heterogeneity							
Clinicopathological features	Number of studies	Number of patients	Pooled OR (95% CI)	*P*_Het_	*I^2^* (%)	*P*-value	Model used
Gender	12	1020	0.88 [0.67, 1.15]	0.992	0.0	0.992	Fixed
Age	12	1091	1.19 [0.93, 1.51]	0.997	0.0	0.336	Fixed
Tumor size	9	801	2.10 [1.56, 2.84]	0.172	0.0	<0.001	Fixed
Differentiation	9	812	1.45 [0.73, 2.88]	<0.001	73.0	0.283	Random
Lymph node metastasis	7	629	2.26 [1.22, 4.22]	0.006	67.1	0.010	Random
TNM stage	8	658	0.44 [0.32, 0.60]	0.614	0.0	<0.001	Fixed

Abbreviations: Fixed, fixed-effects model; Random, random-effects model.

## Discussion

Many recent studies have indicated that lncRNA FOXD2-AS1 may play critical roles in the progression and development of cancers. FOXD2-AS1 may be involved in progression of tumors through sponging of tumor-suppressive microRNAs. Zhu et al. [38] proposed that FOXD2-AS1 could competitively sponge miR-185-5p to affect the expression of cell division control protein 42 (CDC42), suggesting that CDC42 is a potential downstream molecule of FOXD2-AS1 in CRC and that the complex axis of FOXD2-AS1/miR-185-5p/CDC42 modulated the proliferation and invasion of CRC. In accordance with this finding, Shen et al. [29] reported that FOXD2-AS1 regulated the malignancy of glioma via the FOXD2-AS1/miR-185-5p/CCND2 axis. Moreover, in glioma, Dong et al. [36] found that FOXD2-AS1 can act as an endogenous sponge of miR-185, which can bind AKT1 to promote cell proliferation and metastasis. In other cancers, FOXD2-AS1 has been suggested to contribute to migration and invasion of tumors by sponging miR-185-5p [15,28–30,36,38,40,46], miR-25-3p [20], miR-98-5p [47], miR-31 [48], miR-7-5p [49], miR-760 [44], miR-195 [24], miR-145-5p [25], miR-363-5p [13,17], miR-143 [50], miR-485-5p [37], and miR-206 [14]. Recent work has shown that lncRNAs influence the occurrence and development of tumors by regulating gene expression at the transcriptional or post-transcriptional level. Su et al. [31] found that FOXD2-AS1 affects hnRNPL regulation of TRIB3 expression by directly binding to the promoter of TRIB3. In addition, FOXD2-AS1 can form a positive feedback loop with AKT and E2F1 to affect the malignant phenotype of bladder cancer. FOXD2-AS1 can also regulate EMT-related proteins by activating signal pathways such as the Notch [19], Wnt/β-catenin [21], Hippo signaling pathway, mTOR, MAP3K1 and PI3K/AKT [31] pathways. And the meta-analysis demonstrated that the expression of FOXD2-AS1 was associated with the tumor size and lymph node metastasis, which may be contributed by which FOXD2-AS1 function as a oncogenic role on the proliferation, migration, invasion in cancers. The mechanisms of action for FOXD2-AS1 as described in published articles are summarized in Table 6.

**Table 6:** Summary of FOXD2-AS1 with their potential targets, pathways and related microRNAs

Cancer type	Expression	Functional role	Related microRNAs	Downstream molecules	Protein binding	Signaling pathway
Colorectal cancer [19,20,38]	up-regulation	Cell proliferation, migration, invasion, EMT	miR-185-5p/miR-25-3P	CDC42/sema4c	/	Notch signaling pathway
Bladder cancer [31,50]	up-regulation	Tumor growth, accelerate the gemcitabine-resistance	miR-143	ABCC3	/	/
NSCLC [21,22]	up-regulation	Cell growth and tumor progression; cisplatin resistance	miR-185-5P	β-catenin/TCF/SIX1	/	Wnt/β-catenin signaling
NC [13]	up-regulation	Cell growth	miR-363-5p	S100A1	/	/
Glioma [27–30,36,46–48]	up-regulation	Cells proliferation, migration, invasion and EMT, and promoted apoptosis; drug resistance	miR-185/miR-185-5 p/miR-98-5p/miR-31	AKT1/CCND2/p53/ CPEB4/CDK1	GREB1	PI3K/AKT
Gallbladder cancer [51]	up-regulation	Cell proliferation, migration, and invasion	/	MLH1	/	/
CM [33,52]	up-regulation	Cell proliferation, migration, and invasion	/	/	p-AKT	/
GC [18]	up-regulation	Cell growth, cell cycle	/	E2F1/E2F2/CDK4/ EphB3/PCNA	EZH2/LSD1	/
Papillary thyroid cancer [37,40]	up-regulation	cell proliferation, migration and induce cell apoptosis	miR-485-5p/miR-7-5p	KLK7/TERT	/	/
HCC [14–17,42]	up-regulation	Cell viability and metastasis;resistance to sorafenib	miR-206/miR-185/miR-150-5p	ANXA2/CDKN1B	EZH2/DKK1	Wnt/β-catenin signaling MAP3K1/AKT
Breast cancer [26,53]	up-regulation	Cell growth, cell cycle	miR-150-5p	PFN2	S100	Hippo signaling pathway
Cervical cancer [39,44]	up-regulation	Cell proliferation, migration	miR-760	HDGF	CDX1	/
Esophagus cancer [25]	up-regulation	Cell viability and invasion	miR-145-5p/miR-195	CDK6	/	AKT/mTOR
		Cisplatin resistance				
Laryngeal squamous cell [54]	up-regulation	Chemothrapetutic resistance	/	STAT3	/	/

In addition to exploring the molecular mechanisms of lncRNA FOXD2-AS1, recent studies have also investigated FOXD2-AS1 as a tumor-specific biomarker. Because most previous studies have been limited by small sample size, we performed a comprehensive meta-analysis and TCGA data review. The results demonstrated that the high expression of FOXD2-AS1 was correlated with advanced clinicopathological features such as tumor size and TNM stage. Moreover, the pooled HRs indicated a significant relationship between FOXD2-AS1 and poor OS, and the subgroup analysis indicated FOXD2-AS1 was related to poor OS for different sources and sample size. However, the expression of FOXD2-AS1 was only correlated with poor OS in digestive tumors, and was not correlated in respiratory, female reproductive and nervous system tumors, suggesting that the mechanism of FOXD2-AS1 may be different in various tumors. When we conducted subgroup analysis based on region, FOXD2-AS1 expression was related to poor OS only in Asian population, but not in American and other Western countries, which suggests that FOXD2-AS1 may be only suitable as a biomarker in the Asian population. We next explored the prognostic value of FOXD2-AS1 in the TCGA dataset, and the results indicated that high expression of FOXD2-AS1 was related to poor OS and DFS in 9502 patients. When we assessed the role of FOXD2-AS1 in different tumor types, FOXD2-AS1 was associated with poor OS in hepatobiliary and pancreatic, urinary, and head and neck cancers, but not in respiratory system tumors. Similarly, FOXD2-AS1 was related to poor DFS in urinary tumors, respiratory tumors, and head and neck tumors. Interestingly, the expression of FOXD2-AS1 was related to favorable prognosis in GI and female reproductive tumors, but more studies are needed to verify the mechanism of FOXD2-AS1 action in various tumors.

Some limitations of the present study should be emphasized. First, all of the included articles are from China, so the conclusions may be only applicable to a Chinese or Asian population. However, we also analyzed data from the GEO database and TCGA dataset. There were some differences in the meta-analysis and analysis of the TCGA dataset. In particular, the meta-analysis reported FOXD2-AS1 was related to poor OS in digestive tumors, however, the TCGA data indicated the opposite result. This may reflect differences in the study populations. Overall, studies should include more patients from different regions. Second, some studies, which have not been published, may influence the publication bias. Third, in some articles, the specific HR value was not provided, and we had to extract the HR value from the K–M curve, a process that may introduce some errors. Fourth, the sample size and tumor types included in this analysis are still limited. Fifth, different cut-off values for up-regulated expression of FOXD2-AS1 were applied in these studies, which may contribute to the data heterogeneity.

Although this article has some limitations, the results are meaningful. The pooled results indicate that the high expression of lncRNA FOXD2-AS1 is associated with larger tumor size and advanced TNM stage. FOXD2-AS1 was also related to a poor OS and DFS in solid tumors, so FOXD2-AS1 may be a potential prognostic biomarker for patients with cancers. However, the role of FOXD2-AS1 may vary in various tumor types, race and regions, so more high-quality datasets and articles with large sample size are needed to verify the role of FOXD2-AS1 in different tumor types and regions.

## Abbreviations

CDC42: cell division control protein 42
CI: confidence interval
CLL: chronic lymphocytic leukemia
DFS: disease-free survival
ESCC: esophageal squamous cell carcinoma
FOXD2-AS1: FOXD2 adjacent opposite strand RNA 1
GC: gastric cancer
GI: gastrointestinal
HCC: hepatocellular carcinoma
HR: hazard ratio
lncRNA: long non-coding RNA
NC: nasopharyngeal carcinoma
NOS: Newcastle–Ottawa Scale
NSCLC: non-small cell lung cancer
OR: odds ratio
OS: overall survival
TCGA: the Cancer Genome Atlas
